# A Case of Primary Breast Diffuse Large B-Cell Lymphoma Treated with Chemotherapy Followed by Elective Field Radiation Therapy: A Brief Treatment Pattern Review from a Radiation Oncologist's Point of View

**DOI:** 10.1155/2015/907978

**Published:** 2015-07-13

**Authors:** Kyu Chan Lee, Seung Heon Lee, KiHoon Sung, So Hyun Ahn, Jinho Choi, Seok Ho Lee, Jae Hoon Lee, Junshik Hong, Sang Hui Park

**Affiliations:** ^1^Department of Radiation Oncology, Gil Medical Center, School of Medicine, Gachon University, Incheon 405-760, Republic of Korea; ^2^Department of Internal Medicine, Gil Medical Center, School of Medicine, Gachon University, Incheon 405-760, Republic of Korea; ^3^Department of Pathology, Ewha Womans University School of Medicine, Seoul 158-710, Republic of Korea

## Abstract

We here report a case of primary breast lymphoma (PBL). A 44-year-old woman presented with a painless mass in the right breast. Fine needle aspiration cytology and excisional biopsy were performed. Excisional biopsy revealed low grade lymphoma, which was subsequently confirmed with histopathology and diagnosed as diffuse large B-cell lymphoma (DLBCL). A chest computed tomography scan revealed a 3.5 cm sized breast mass with skin thickening and a small sized lymphadenopathy in the ipsilateral axilla. Radiation therapy including the right whole breast and ipsilateral axilla and supraclavicular lymph node was performed after the patient received four courses of R-CHOP (cyclophosphamide, doxorubicin, vincristine, and prednisolone plus rituximab) chemotherapy. At the follow-up period of 42 months, the patient is surviving with no evidence of disease. No morbidities occurred in this patient during the follow-up period. We also briefly review the current practice pattern in PBL patients with DLBCL.

## 1. Introduction

Primary breast lymphoma (PBL) is a rare tumor that originates from lymph tissues. The reported incidence is 0.04–0.5% of malignant breast tumors [1]. The incidence of PBL in all non-Hodgkin's lymphoma (NHL) cases is less than 1%. And the most common histology in PBL was diffuse large B-cell lymphoma (DLBCL) [2]. Although PBL may behave in a similar clinical and radiological presentation as breast carcinoma, treatment modalities and outcomes differ. Because of its rarity, the treatment approach varies greatly. Although the use of radiation therapy (RT) and/or chemotherapy (CTx) differs in the literature, combined therapy with surgery, CTx, and involved field radiation therapy (IFRT) or elective field radiation therapy (EFRT) is currently considered to be the standard treatment approach for PBL patients with DLBCL [2, 3].

However, confidential data regarding appropriate treatment strategy including RT techniques such as RT field, RT dose, and RT fraction size of DLBCL in PBL are still lacking. Here, we present a case of PBL treated with chemotherapy followed by EFRT and also performed a brief literature review of the current practice pattern of PBL patients with DLBCL.

## 2. Case Report

A 44-year-old woman was admitted to our hospital with a mass in the right breast. A physical examination revealed a hard mass in the upper outer quadrant (UOQ) of the right breast, located approximately 2 cm from the nipple. The exam also revealed a nontender mass that was about 3 cm in size. The contralateral breast was normal. The patient had no previous history of liver cirrhosis, hepatitis B infection, and diabetes mellitus. The ethnicity of patient was East Asian. A physical examination of the neck and axilla was negative for enlarged lymph nodes. Beta2-microglobulin level was 890.4 ng/mL and the lactate dehydrogenase (LDH) level was 390 IU/L. The patient had no B symptoms (fever, weight loss, or night sweats).

**Figure 1:**
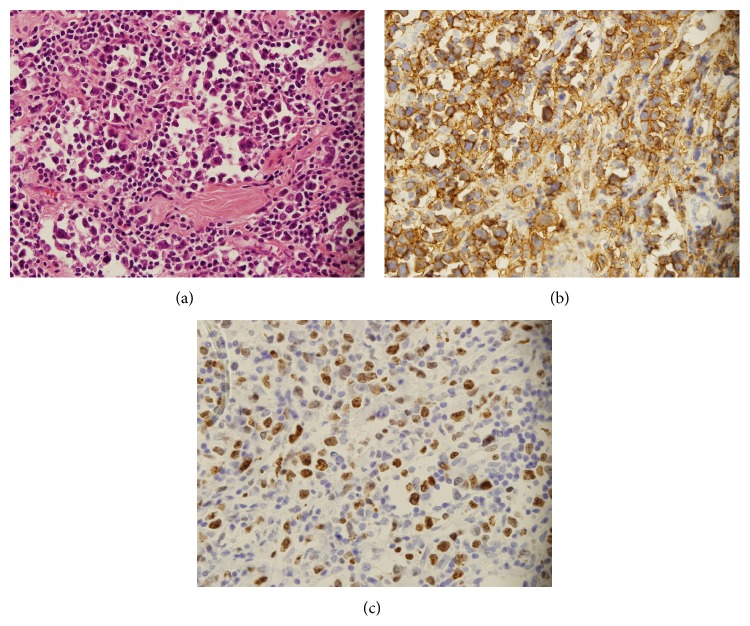
Histopathological examination shows diffuse dense infiltration of small and large lymphoid cells (a, ×400), composed of predominantly CD20 positive cells (b, ×400). Excisional biopsy revealed low grade lymphoma subsequently confirmed by histopathology and diagnosed as diffuse large B-cell lymphoma. CD 20+/CD 30−/CD 3−/cyclin D1- and index of proliferation Ki-67 positive for tumor cells (c).

Fine needle aspiration cytology (FNAC) and excisional biopsy were performed. FNAC showed small lymphocytic infiltration with some large atypical cells and recommended additional ancillary test such as immunochemistry or excisional biopsy for confirmative diagnosis. Therefore, an excisional biopsy was performed and showed atypical lymphocytic infiltration suspicious for lymphoid malignancy. Additional immunohistochemical stains were performed for a confirmative diagnosis. CD20 and Ki-67 expression were positive by immunochemistry (Figure 1). Finally, excisional biopsy revealed low grade lymphoma, which was subsequently confirmed by histopathology and diagnosed as DLBCL.

**Figure 2:**
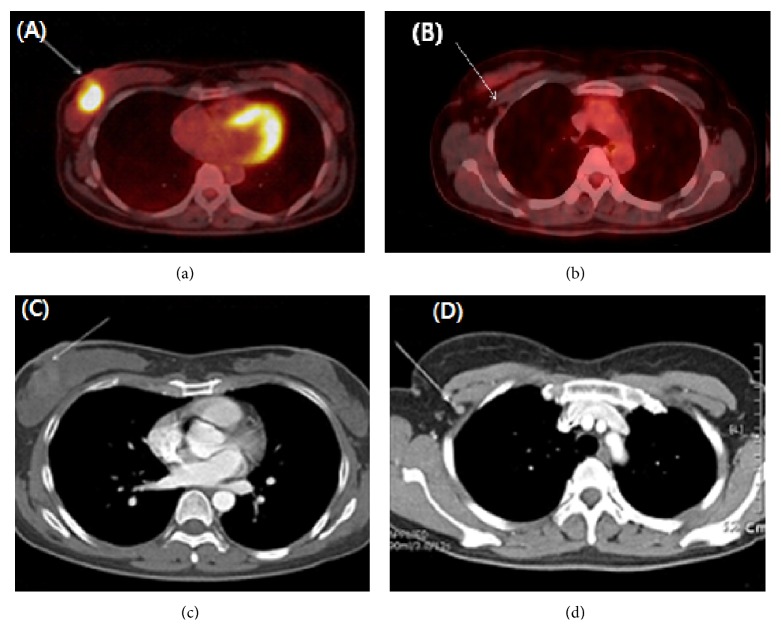
F-18 fluorodeoxyglucose positron emission tomography/computed tomography (CT) reveals a hypermetabolic lesion (arrows) in the right breast (a) with mild hypermetabolic uptake in the ipsilateral axilla (b) and about a 3.5 cm sized right breast mass (c) with skin thickening. A small (7 mm) sized lymphadenopathy is observed in the ipsilateral axilla (d) on a plain contrast CT scan of the chest.

Chest and abdominal computed tomography (CT) and positive emission tomography (PET) scans were evaluated for the staging workup. A bone-marrow (BM) biopsy was also performed. The chest CT revealed a 3.5 cm sized breast mass with skin thickening and a small (7 mm) sized lymphadenopathy in the ipsilateral axilla. A PET scan showed hypermetabolic uptake in the UOQ of the right breast with mild hypermetabolic uptake in the ipsilateral axilla (Figure 2). The patient was diagnosed with DLBCL. The BM biopsy showed a negative result for lymphomatous infiltration. The patient was diagnosed with stage IEA primary breast lymphoma according to the Ann Arbor staging system.

**Figure 3:**
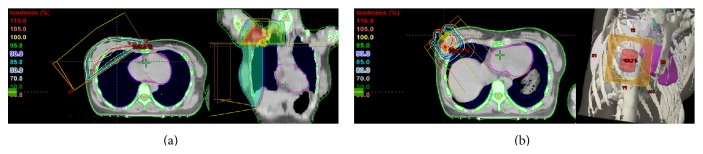
The patient received elective field radiation therapy including the whole right breast, ipsilateral axilla, and supraclavicular lymph node (a) with dose prescription of 3,600 cGy in 20 fractions over 4 weeks plus a local boost (b) of 1,440 cGy to the primary site in 8 fractions over 1 week.

The patient received four courses of R-CHOP (cyclophosphamide, doxorubicin, vincristine, and prednisolone plus rituximab) CTx. After two courses of R-CHOP CTx, the follow-up chest CT showed decreased size of the right breast mass (3.5 cm → 2 cm) and right axillary lymph node (7 mm → 3 mm). After four courses of R-CHOP CTx, the follow-up chest CT showed no visible mass in the breast or axilla. The EFRT including the right whole breast, ipsilateral axilla, and supraclavicular lymph node (SCLN) was performed. An RT dose of 36 Gy in 1.8 Gy daily fractions was given to the whole right breast, ipsilateral axilla, and SCLN. After whole breast RT, a boost to the primary tumor bed was performed with a direct 12 MeV electron beam at a dose of 14.4 Gy. The primary tumor bed was irradiated with a total dose of 50.4 Gy in 28 fractions (Figure 3).

Follow-up chest CT and breast ultrasonography (USG), performed 32 months after RT, were normal. At a follow-up period of 42 months, the patient is surviving with no evidence of disease. No morbidities (such as radiation pneumonitis or arm edema) occurred in this patient during the follow-up period.

## 3. Discussion

The rarity of this cancer is because the breast contains less lymphoid tissue than other organs, such as the intestines and lungs, where primary lymphomas are more common [4]. PBL was traditionally defined as localized lymphoma to one or both breasts with or without regional lymph nodes such as ipsilateral axillary and/or SCLNs [5]. The most common pathological diagnosis in PBL is DLBCL. The following should also be included in the differential diagnosis of PBL: primary breast cancer, inflammatory breast cancer, fibroadenoma, phyllodes tumor, pseudolymphoma, metastatic disease, and benign breast neoplasm [6–8]. DLBCL is the most common histopathological type of PBL. The other frequent histological types are follicular lymphoma (15%), mucosa associated lymphoid tissue lymphoma (12.2%), Burkitt's lymphoma, and Burkitt-like lymphoma (10.3%) [9].

Although the present case was diagnosed with PBL in the fifth decade, the peak age for PBL is usually the sixth decade. The peak age of PBL is different between ethnicities. The median age in Western countries is over sixty years (62–64 years), whereas the median age in East Asian countries (45–53 years) is lower [10]. The right breast was involved in our case. PBL occurs more frequently in the right breast, with a 3 : 2 ratio [3, 11]. Breast USG was not performed in our case after biopsy results were obtained. Instead, chest CT and PET scans were conducted. The CT features in our study were circumscribed or ill-defined masses with homogenous, heterogeneous, or rim enhancement. Because none of these imaging features of PBL are pathognomonic, a biopsy was performed. Not only a histopathological examination but also immunophenotyping is an effective way to confirm aspiration cytology findings. In this report, we performed not only aspiration cytology but also excisional biopsy, and the histopathological examination showed diffuse infiltration of the small and large lymphoid cells, which were composed predominantly of CD20 positive cells.

No consensus exists on the best way to treat PBL, but the current treatment modality is considered as combined therapy consisting of CTx and RT. It has been previously reported that CTx with CHOP regimens and RT can significantly increase the overall survival and progression-free survival compared to CTx alone in localized intermediate- and high-grade NHL. Miller et al. concluded that CTx with three cycles of CHOP regimen followed by IFRT is superior to CTx with eight cycles alone [12]. Nowadays, the most common chemotherapeutic regimen for treating PBL is the R-CHOP regimen. In particular, rituximab, a monoclonal antibody targeting the CD20 antigen, is reported to have high efficacy for DLBCL [13]. The use of rituximab for CHOP CTx has been reported to reduce the overall CNS relapse risk in PBL patients with DLBCL [14].

We briefly reviewed the role of surgery, CTx, and RT in PBL patients with DLBCL as follows. Initially, most patients received surgery ranging from lumpectomy to mastectomy [9]; however, most studies have been inconclusive regarding the effects of surgery on PBL. The study reported by Ryan et al. [3] showed that surgeries including mastectomy were associated with increased risk of mortality. And this study of 204 patients with PBL showed that there was no benefit from mastectomy, as opposed to biopsy or lumpectomy. Even axillary node dissection did not show survival benefits [11]. They reported that the combination of limited surgery, anthracycline-based CTx, and IFRT produced the best outcome in the pre-rituximab era and concluded that combined therapy was the best method for treating patients with PBL [3]. They also suggested prophylactic therapy for avoiding central nervous system (CNS) involvement. It is now well established that limited surgery should be performed for diagnostic purposes for PBL and should not be considered a treatment modality. The CTx, especially with an anthracycline-based regimen which showed positive effects on overall survival, is a main treatment component of PBL patients with DLBCL [3]. The addition of rituximab to the CHOP regimen was also evaluated by several studies [13–15]. Avilés et al. [15] reported that there were no CNS relapses in patients treated with rituximab, whereas a relapse rate of 11% occurred in patients treated without rituximab. The ultimate goal of RT is to consolidate the primary lesions after CTx. The CTx followed by RT has shown a significantly improved survival benefit compared to CTx or RT alone [3, 9, 15]. However, the appropriate RT volume in PBL with DLBCL has not yet been settled. Although initial RT techniques encompassed the ipsilateral breast with or without regional nodes and even contralateral breast in some patients, the current RT approach is to minimize the RT volume into the involved site [16]. The involved site RT technique (IFRT) encompasses the pre-CTx tumor volume with enough margins to include the subclinical disease, setup margin, and so forth. In our case, the EFRT including prophylactic RT to SCLN was performed, which is somewhat different from the IFRT technique. In EFRT, the prophylactic RT to uninvolved nodes is used. We summarized the recent literatures published after the year 2005 on treatment modalities for the PBL patients with DLBCL in Table 1. Avilés et al. reported that a complete response (CR) in patients treated with RT, CTx, and RT and CTx arm was 66.7% (20/30), 59.4% (19/32), and 88.2% (30/34), respectively (*P* < 0.01). They also reported that the most common site of recurrence was the CNS (11.4% (11/96)) and that the acute toxicity was mild [17]. To improve the treatment outcome, the combination CTx that includes a high dose of methotrexate and/or Ara C for CNS prophylaxis should be considered for patients with aggressive forms of PBL, even in the early stage [18].

In the present study, the patient received the R-CHOP regimen followed by RT. Although the CR of the breast tumor after treatment of a four-cycle R-CHOP had been achieved, we then hypothesized that the ipsilateral whole breast, axilla, and SCLN may have the potential risk of subclinical disease. Therefore, we planned to treat the patient with EFRT including not only the whole right breast and ipsilateral axilla, but also SCLN. Because an axillary lymph node biopsy could not be performed for a pathological confirmation due to the deep-seated location, the initial stage before starting CTx was IEA according to the Ann Arbor staging system. However, we considered this patient could be staged as IIEA (limited to the breast and ipsilateral axilla) because axillary metastasis was strongly suspected considering the axillary response to CTx. One report indicates that the EFRT policy for PBL should include the breast, ipsilateral axilla, and supraclavicular region in RT fields [2]. Modern RT technology such as intensity modulated radiation therapy and respiratory gating techniques will further decrease the RT dose to normal tissues and the risk of RT related toxicities.

Although there are controversies regarding the prognostic factors in patients with PBL, a favorable international prognostic index (IPI) score, use of anthracycline-containing CTx, and RT have been reported as significant prognostic factors for longer overall survival (OS) [3]. The patient in our report had an initial IPI score of 0-1 and received anthracycline-containing CTx followed by RT. Thus, more favorable survival results may have been expected in our patient.

## 4. Conclusion

Here, we report a case of PBL with a review of the literature. The PBL was confirmed by histopathology and immunohistochemistry and treated with EFRT after CTx. We suggest that RT is an effective and safe option and combined CTx and EFRT can give a longer survival in PBL cases with axillary lymph node involvement.

## Figures and Tables

**Table 1:** Brief review of treatment modalities and results in PBL patients with DLBCL.

Author [Ref]	*N*	Age (median)	Treatment modality						Results			
			Surgery (%)	CTx		RT (%)			CR (%)	CNS relapse (%)	Survival (%)	
				CHOP (%)	Rituximab (%)	*N* (%)	Field	RT dose (Gy/Fx)			5 Y-OS	PFS
Avilés et al. [17]	96	58	0	66 (69)	0	64 (67)	IFRT	45/20	69 (72)	11 (11.5)	76 (10 Y)	83 (10 Y)
Yoshida et al. [19]	15	68	11 (73)	12 (80)	0	NA	NA-	NA	NA	0	NA	NA
Avilés et al. [15]	32	46	NA	32 (100)	32 (100)	32 (100)	IFRT	45/20	28 (87)	0	63 (3 Y)	75 (3 Y)
Salzberg et al. [20]	75	62	NA	68 (91)	52 (69)	51 (68)	NA	NA	NA	10 (13.3)	75	65
Mouna et al. [21]	7	50	4 (57)	6 (86)	2 (29)	2 (29)	EFRT	36–50	7 (71.4)	0	NA	NA
Seker et al. [22]	9	49	0	8 (89)	7 (78)	5 (56)	IFRT, EFRT	NA	7 (77.7)	0	76.2	NA
Yhim et al. [5]	68	48	23 (34)	66 (97)	42 (62)	21 (31)	NA	NA	54 (83.1)	0	60.7	50.3
Niitsu et al. [23]	30	57	NA	30 (100)	11 (37)	18 (60)	IFRT	NA	29 (96.7)	2 (6.7)	87	77
Zhao et al. [24]	1	39	1 (100)	1 (100)	1 (100)	1 (100)	IFRT	40/NA	100	0	NA	NA
Validire et al. [25]	38	62	NA	36 (95)	4 (10)	27 (71)	IFRT, CNS PRT	40/20 18/10	34 (89)	3 (6.7)	61	54
Present study	1	44	no	1 (100)	1 (100)	1 (100)	EFRT	50.4/28	100	0	NA	NA

Ref: references; *N*: total number of patients; NA: not applicable; Gy: Gray; Fx: fractionation; CTx: chemotherapy; RT: radiation therapy; 5 Y-OS: 5-year overall survival; PFS: progression-free survival; CR: complete response; IFRT: involved field radiation therapy; EFRT: elective field radiation therapy; CNS PRT: central nervous system prophylactic radiation therapy.
